# Understanding factors influencing questionnaire response rates to maximise retention in a long term complex intervention trial

**DOI:** 10.1186/1745-6215-12-S1-A134

**Published:** 2011-12-13

**Authors:** Athene J Lane, Liz Down, Julia Wade, David Neal, Freddie Hamdy, Jenny Donovan

**Affiliations:** 1 School of Social and Community Medicine, University of Bristol, Bristol, BS8 2PS, UK; 2 University Dept of Oncology, Addenbrooke’s Hospital, Cambridge, CB2 0QQ, UK; 3 Nuffield Dept of Surgical Sciences, University of Oxford, Oxford, OX3 9DU, UK

## Objectives

Participant retention is challenging in long term trials and attrition can threaten trial validity, especially for patient-reported outcomes which cannot be ascertained otherwise. The evidence base to maximise retention is limited so we investigated factors influencing questionnaire response rates with a mixed methods research approach.

## Methods

ProtecT is an ongoing randomised trial of localised prostate cancer treatments in men currently aged 53-76 years (ISCRTN 20141297) with six years follow-up. Symptoms and QoL were collected annually by postal questionnaire with a reminder letter for non-responders at six weeks (phase I). Three additional interventions were sequentially investigated to increase retention. Firstly, clinical centre staff commenced telephoning non-responders at four weeks (phase II). A study pen was included with reminder letters and a shortened questionnaire was sent to non-responders at nine weeks (phase III). Questionnaire response rates over six months were compared by phase. 1279 participants were asked in the questionnaires about online completion. 18 participants gave in-depth interviews about trial follow-up which were transcribed verbatim and analysed thematically.

## Results

The questionnaire response rate was 76.4% (phase I: 1045/1367) with a median return of 13 days (IQ range 8-22 d) and the reminder letter increased this to 86.8% (1187). Telephoning increased rates to 90.5% (phase II: 1105/1221). The additional incentive of a study pen was ineffective (phase III: 1026/1142, 89.8%) whilst the short questionnaire had some impact (phase III: 9/84 posted, 10.7%, 1033/1142, 90.5%). One quarter of men wished to complete on-line questionnaires (430, 25.2%).

In interviews, most men found questionnaires acceptable and understood their purpose although they were regarded as a less enjoyable aspect. Some men saw questionnaires becoming less relevant over time either because they felt they were cured or they had become repetitive, although they continued to complete them. Participant newsletters were interesting and gave a sense of belonging to a group although some men wanted preliminary trial findings. The study website was infrequently accessed (four men did not use the web), partly because men assumed it contained no extra information.

## Conclusions

A reminder letter and telephone call increased questionnaire response rates in a trial with older men. A study pen provided no incentive but response rates were already 90%. Men understood the purpose of questionnaires but some attitudes could increase attrition and need to be addressed by trialists. Newsletters maintained participant interest, unlike the website.

The ProtecT study is funded by the Health Technology Assessment programme.

